# The Clapham Maternity Hospital

**Published:** 1889-11-02

**Authors:** M. Ritchie

**Affiliations:** Hon. Sec.


### THE CLAPHAM MATERNITY HOSPITAL.

I see in The Hospital of Sept. 28 a friendly notice by "A Clerical Correspondent" of the Clapham Maternity Hospital recently opened. While thanking him for his appreciative remarks, may I make a few corrections on points which, as it seems to me, he has somewhat misunderstood. The Clapham Maternity Charity was originally begun with the idea of giving the advantages of improved midwifery to a class of women too poor to pay a doctor and who are usually in the hands of ignorant and uncertificated midwives. Later, a second *raison d'etre* for the institution arose from the fact of a School of Medicine for Women, numbering about 90 students, existing in London, with no other means of training its students in practical midwifery as medical students, and not merely midwives, except those afforded by the small maternity work here. About 60 students have been trained during the last four years. 2nd. We should like it to be understood by all taking any interest in the matter that the institution is worked under an auditing committee, and that *all* the payments of patients, out-patients as well as in, are made to the *funds of the institution*, which are supplemented by subscriptions, while the medical work of the doctors in charge is honorary. May I add that the Dispensary for Women and Children, purposely kept apart from the Hospital itself, is at 2, Stockwell-road—the address given by your correspondent was accidentally incorrect.

M. Ritchie, Hon. Sec.

A Word about Whisky.—We have already referred to Messrs. Ferguson's, of Reading, old Scotch "M. D." whisky. With all deference to the analysts, we would submit that "The Test of good whisky is in the drinking." An experienced taster, not a tippler, will give a better decision as to the presence or absence of fusel oil, the fineness of the aroma, the maturity of the spirit, and other qualities than even the analyst himself. To three such experienced tasters, two of them Scotch, and all of them possessed of palates unspoilt by regular drinking, even in small quantities, we have submitted samples of the "M. D." whisky sent us by Messrs. Ferguson. They all pronounced it "decidedly good, fine and mellow for toddy, and mild for use as a stimulant for the sick." If to this practical proof the results of a chemical analysis be added, drinkers of whisky will have all the assurance they need that the Messrs. Ferguson can supply them with an excellent brand.

